# Donor support for Health Policy and Systems Research: barriers to financing and opportunities for overcoming them

**DOI:** 10.1186/s12992-022-00896-4

**Published:** 2022-12-23

**Authors:** Alexander Kentikelenis, Abdul Ghaffar, Martin McKee, Livia Dal Zennaro, David Stuckler

**Affiliations:** 1 Department of Social and Political Sciences, Bocconi University, via Roentgen 1, 20136 Milano, Italy; 2 Alliance for Health Policy and Systems Research, World Health Organization, Geneva, Switzerland; 3 London School of Hygiene and Tropical Medicine, London, UK

**Keywords:** Health Policy and Systems Research (HPSR), Global health financing, Donor priorities, Health Systems

## Abstract

**Background:**

The vast investments that have been made in recent decades in new medicines, vaccines, and technologies will only lead to improvements in health if there are appropriate and well-functioning health systems to make use of them. However, despite the growing acceptance by major global donors of the importance of health systems, there is an enthusiasm gap when it comes to disbursing funds needed to understand the intricacies of how, why and when these systems deliver effective interventions. To understand the reasons behind this, we open up the black box of donor decision-making vis-à-vis Health Policy and Systems Research (HPSR) financing: what are the organizational processes behind the support for HPSR, and what are the barriers to increasing engagement?

**Methods:**

We conducted 27 semi-structured interviews with staff of major global health funders, asking them about four key issues: motivations for HPSR financing; priorities in HPSR financing; barriers for increasing HPSR allocations; and challenges or opportunities for the future. We transcribed the interviews and manually coded responses.

**Results:**

Our findings point to the growing appreciation that funders have of HPSR, even though it is often still seen as an ‘afterthought’ to larger programmatic interventions. In identifying barriers to funding HPSR, our informants emphasised the perceived lack of mandate and capacities of their organizations. For most funding organisations, a major barrier was that their leadership often voiced scepticism about HPSR’s long time horizons and limited ability to quantify results.

**Conclusion:**

Meeting contemporary health challenges requires strong and effective health systems. By allocating more resources to HPSR, global donors can improve the quality of their interventions, and also contribute to building up a stock of knowledge that domestic policymakers and other funders can draw on to develop better targeted programmes and policies.

**Supplementary Information:**

The online version contains supplementary material available at 10.1186/s12992-022-00896-4.

## Background

There is widespread consensus that health systems strengthening is critical for achieving health and development goals [1, 2]. The vast investments that have been made in recent decades in new medicines, vaccines, and technologies will only lead to improvements in health if there are appropriate and well-functioning systems to make use of them. These discussions have been thrown into sharp relief by the Covid-19 pandemic and its aftermath: countries’ ability to respond to the health crisis and roll out vaccines in part depend on the presence of an effective and well-equipped health system that can cater to the needs of the population [3–5].

However, despite the growing recognition of health systems in the portfolios of major global donors [6–8], there is an enthusiasm gap in terms of disbursing funds to understand the intricacies of how, why and when these systems deliver effective interventions. That is, research into health policy and systems suffers from chronic underfunding, notwithstanding its potential to improve the effectiveness and efficiency of health interventions [9]. One recent analysis of $246 billion in committed global health and population development projects between 2000 and 2014 found that only about $4 billion—or 2%—was allocated to Health Policy and Systems Research (HPSR) [10].

There are multiple reasons why global donors may neglect HPSR. One is a potential lack of clarity regarding its scope and nature [11]. There is considerable confusion between fields such as ‘health systems research’ and ‘health services research’, and how HPSR distinguishes itself from related areas of ‘implementation research’, ‘evidence-based policymaking’ or ‘operations research’ (see Table 1 for definitions). Second, HPSR tackles ‘difficult’ issues that funders may not want to confront, such as partisan politics or corruption [12]. Third, sometimes there is a lack of structures, like think tanks and research institutes, in recipient countries to ensure that findings from HPSR translate into policy changes [13, 14]. Finally, HPSR is often context dependent, whereas funders tend to look for generalisable solutions. This is particularly the case in the search by some funders for a ‘silver bullet’ or for taking a ‘big bet’ [15]. This can oversimplify challenges and favour health technology solutions, like the development of a treatment, while neglecting the broader health system context that can impact whether any proposed solutions will work.

**Table 1:** Multiple definitions and visions of HPSR

Health Policy and Systems Research (HPSR) focuses on “how societies organize themselves to achieve health goals” [16]. This research remit entails generating new and reliable knowledge on health policy and systems, and promoting its application in health interventions by competent policymakers [17]. In this context, strengthening domestic research capacities to undertake HPSR and expanding opportunities to feed this research into the policy process is essential for delivering effective and sustainable improvements in health [17].
Operations research refers to the production of knowledge that can be used to resolve problems in health interventions [18].
Implementation science refers to the study of how to improve the uptake, implementation and translation of research findings into routine practices [18].

In this article, we open up the black box of donor decision-making vis-à-vis HPSR financing: what are the organizational processes behind the growing support for HPSR, and what are the barriers to increasing engagement? Answering these questions is an important step towards understanding the conditions under which HPSR can increase in countries that receive significant development assistance for health, and the ways in which donors can be prompted to channel more financing to this end.

## Methods

To answer our research questions, we conducted interviews with staff of major global health funders during the first quarter of 2021. We identified potential informants through a search of open calls for HPSR funding uploaded on the devex.com platform, suggestions by the World Health Organization (WHO) Alliance on Health Policy and Systems Research, and the recommendations of initial interviewees. This meant that all informants worked for many of the largest global health funders (e.g., donor agencies from countries in the Global North that extensively fund development assistance for health, large multilateral organizations with a remit to finance health interventions, and major philanthropic foundations). By design, our interviews did not extend to funders of health-related research who have a domestic remit.

In total, we invited 38 individuals, 27 of whom responded positively as summarized in Table 2. Interviews generally lasted between 30 and 60 min and were granted on the condition of anonymity. The number of interviews was determined by two factors: ensuring a broad representation of different types of global health funders (multilateral, bilateral, non-governmental); and reaching the point of data saturation (i.e., when we reached the point where we had interviewed professionals from diverse global funders and no major new themes emerged in the interviews). Given our substantive interest in centralized decisions over HPSR funding, all but one interviewee were based in the headquarters of their organizations.

**Table 2:** Summary of interviewees

Type of funder	Interviews	Example organizations
Bilateral	10	US National Institutes of Health (NIH); German Ministry of International Cooperation and Development (BMZ); UK Foreign, Commonwealth and Development Office (FCDO)
Multilateral	3	Global Fund; European Commission
Non-governmental	12	Bill & Melinda Gates Foundation; Doris Duke Charitable Foundation; Open Society Foundations
Opinion leaders	2	

The interviews followed a loosely structured questionnaire to enable flexibility and tailoring to the knowledge and experiences of each informant. As reported in Web Appendix 1, we developed a different interview script depending on whether an interviewee’s organization was a consistent supporter of HPSR or not, following the views of the interviewee on this topic. In practice, only one informant reported that their organization was inconsistently supporting HPSR, while all others described their organizations as either consistent in their support for HPSR or rapidly scaling up such activities, in part to deliver on the health systems strengthening element of the global policy agenda. Consequently, we mostly followed the questionnaire for consistent supporters of HPSR.

Informed consent was secured both through the initial e-mail communication with informants and in the opening of each interview where—following explicit agreement—we started recording. Once each interview was completed we transcribed it to enable manual coding of responses. Quotes or opinions cited in the Results section have the prefix M for officials in multilateral donors, B for those in bilateral donors (i.e., development agencies or other governmental organizations in high-income countries), NG for staff of non-governmental organizations (including philanthropic foundations), and O for opinion leaders.

To analyse the interview findings, we relied on inductive reasoning and narrative synthesis. We developed a coding scheme for the interview transcripts on the basis of iterative reading of informants’ answers. Drawing on this inductively-developed scheme, we organize the interview findings around four key themes: motivations for HPSR financing; priorities in HPSR financing; barriers for increasing HPSR allocations; and challenges or opportunities for the future. Together these themes build a broad picture of how major global health funders view HPSR, but also what the major issues are that prevent increasing commitments.

## Results

### Motivations for HPSR financing

Almost all interviewees recognized the importance of HPSR for their line of work. As one informant explained, “[we see HPSR] as a tool to an end” (B10). But this instrumental aspect of HPSR did not mean that funders necessarily saw it as part of their core activities. To the contrary, one senior staffer in a major bilateral donor explained that HPSR “is either a by-product or a prerequisite for our work. We are not a research-funding organization; research comes second to generating policy” (B1). Several informants noted that research components were added mid-way or near the end of projects, as the need for such work to cover gaps within the main project arose (M1, NG2). Sometimes such financing came too late to be able to develop credible research designs (NG10).

In addition to having instrumental interests in supporting HPSR, funders’ motivations also depended on the perceived value they could add to the already-large field of global health financing. When asked about their organization’s role in supporting research projects within the broader health system strengthening agenda, one officer in a large non-governmental organization reflected:

“that space was already well occupied by many other funders, whether global philanthropic foundations or bilateral donors or multilateral banks. And we had some concerns about that agenda and the way it left out marginalized populations and decided that we would place our resources with marginalized populations as they engaged in national-level work around the health system. We came at it from the national grassroots level, rather than engaging with the health system strengthening dialogue that exists at global level” (NG1).

Further, among the emerging motivations of funders—particularly non-governmental ones—was the explicit support of institutions in low- and middle-income countries (NG1, NG3, NG5). For example, one informant commented that their organization has “[an increasing] overall interest in decolonizing global health and trying to support those research bodies and research collectives that are less well-funded in the global research arena” (NG1). Another interviewee explained that they prioritise HPSR projects that are “done in the country, by the country, for the country…we prefer this approach that builds lasting impact, rather than asking for the very best researchers in the world to conduct a study but who are not embedded, which will also mean that policy uptake of findings will be limited” (B5). This could only become a reality if research groups in these countries, which often struggle for institutional funding, received support.

This was not always seen as legally possible, however. One donor stated that their status as a foundation registered in the United States meant that they could only fund US-based institutions. Even so, the donor made every effort to select institutions with long-term partnerships with countries in the Global South: “the bulk of the dollars is actually going to local research institutions in Africa and to their Ministry of Health partnership colleagues, but when one sees the formal grant recipient it is a big US university or research organization” (NG5).

### Priorities in HPSR financing

Once a decision was made to support HPSR, four broad priorities drove funding allocation: generating new data, strengthening capacity, setting up networks, and identifying best practices—we examine each in turn. Importantly, in supporting these areas, many donors pointed out that they actively sought out partnerships with other funding organizations to increase their financing capacity (B5, B6, B9).

#### Data collection

Data collection was seen by large funders as an essential part of their activities, often linked to monitoring and evaluation activities (NG2, NG4, B8, M2). As one official from a large bilateral donor explained, “data collection is supposed to be embedded in everything we do—it’s part of the processes of planning, implementation, assessment, adaptation” (B10). In some cases, data collection was interpreted broadly to also include systematic reviews and operational research (B8).

For most other funders, data collection was limited and tied to specific projects or gaps in knowledge. For instance, an informant from a bilateral funder recalled a project on child health and commented that the country of the recipient organization “didn’t have a national representative survey that it could build upon, so we supported that as part of the project” (B2). Similarly, another informant noted that their organization saw merit in supporting well-targeted HPSR only insofar as they had “a built-in integrated theory of change, or some drive towards social change” (NG7).

#### Capacity strengthening

Capacity strengthening was a key area that absorbed HPSR financing, and this had several dimensions. First, most donors supported *institutional strengthening for organizations and think-tanks* related to health systems and policy. Commonly, this took the form of setting up collaborations with institutions in the Global North, providing research funding or funding for personnel, and building links with policymakers (NG6, B8, OL1). Such capacity-strengthening activities were not limited to supporting the development of research profiles but also were channelled to improving their administrative competences—for example, on good organizational management practices or on grant application skills (NG7, NG11). One research funder reported that they directly identified potential grantee organizations and supported them by providing feedback and guidance on preliminary applications en route to the development of complete applications with high chances of success (NG8).

A second component of capacity strengthening related to *strengthening the skills of individual researchers*. Informants noted the relative lack of research skills vis-à-vis health systems and policy. One interviewee from a major bilateral funder explained that “I was surprised by how hard it was to find people with this skillset. In fact, we ended up having to work with clinical trialists to help them become health policy analysts. I know we have progressed some, but the reality is that this is still one of our big challenges” (B10). In this context, small-scale grants were seen as an especially important way to underpin capacity development for scholars (B3, OL1).

Some funders supported the creation of *cross-national training programmes* or other mentorship initiatives (B4, B5). One informant described how their organization emphasized the creation of mentoring systems, so that researchers and other staff in organizations in the Global South can further develop their skills: “[participants in our training programmes] who may have started off as medical students, then went on to do master’s degrees, and now are heads of department. During the course of our interaction with these participants, we seek to support their future development so that they can get in positions of responsibility” (B6). Even so, funders were wary of solely focusing on strengthening the skills of individuals without associated institutional capacity strengthening : “some of the individuals who got supported to do an MSc or an exchange would then move on to other things, and so the local organization would lose that capacity due to high staff turnover” (NG11).

#### Supporting research networks

Several funders reported supporting the establishment or upkeep of large-scale regional research networks that had a policy orientation—these were often organized around specific policy challenges or priorities. Several respondents who referred to supporting such activities pointed to the importance of these networks being led by and based in countries in the Global South, as this would enable knowledge transfer and the formation of ties between researchers and policymakers within different regions (B6, B7, B9, NG1). For instance, EQUINET, the Regional Network on Equity in Health in East and Southern Africa, was an initiative that sought to bring together scholars, policymakers and civil society to support health system change towards increasing equity—this network received support from several bilateral, multilateral and non-governmental donors.

The support for networks was generally referred to in very positive terms by informants (NG1, B3, B4), noting that—even if policy influence was not the explicit objective—such networks could become long-standing and could build informal or institutionalized links with policymakers (NG1, B4). One of the more developed and well-established cases of network development was the creation of so-called “implementation science alliances,” primarily supported by public US funding. As one informant involved in this process explained,

“The idea was to bring researchers [funded by us] who are doing implementation science-related research in contact with the in-country program implementers who are working in those countries where the research was being done, and with policymakers. So, alliances are comprised of numerous teams. Each team has researchers, program implementers and policymakers who are all in that same ecosystem in a country or a locality. The goal of the alliance is not to necessarily fund the research, because that is already happening, but to support the process of building relationships among these different stakeholders and sectors, and to do two things simultaneously. One, is to inform the research community about what the really primary bottlenecks are on the delivery side. Also, inform the researchers as to what the policy needs were. On the flip side, to sensitize the policymakers to the potential, the promise, but also the limitations of research in terms of what they needed from it or can expect from it, similarly on the program side. Along with those discussions, we integrated training that was tailored not just to the researchers who wanted additional training on research, but also tailored to integrate the policy and program perspective as well” (B4).

Notwithstanding the frequent focus on support for networks in the Global South, some informants noted the potential of high-level networks convened by established universities or organizations. For instance, in addition to their organization supporting two regional partnerships, one interviewee mentioned they also co-financed a commission—led by a prestigious university in the Global North—that sought to draw attention to health systems issues, and set up a network to implement its recommendations (B9).

#### Generating comparative evidence and best practices

A consistent priority for donors was utilizing the findings of HPSR projects to generate evidence on best practices that could travel across different country settings. The underlying motivation was to use HPSR to identify “scalable” interventions that could deliver results across countries in different regions or at different levels of economic development (B4, NG5). Indeed, as one official from a non-governmental donor commented, “we are applying our lessons from our health policy and systems strengthening approaches in Africa to our US grant-making because there are lots of lessons that can travel. In fact, in low- and middle-income settings, because there has been such a history of investment in primary healthcare systems, there is actually considerable capacity” (NG5).

One a major foundation described current attempts to systematize these best practices more clearly. As a senior official explained, “we now support creating what we call ‘exemplars’—case studies of countries that have shown to be able to produce more results for less money. We need to better understand what is happening in these countries, and we have distinct grounds to go and look at their health systems” (NG9).

### Barriers to increasing HPSR financing

We identified four broad categories of barriers to HPSR: related to the funder mandate and capacities, related to recipients of funding, and inherent to HPSR. We examine each in turn.

#### Funder mandate

A recurrent theme raised by informants concerned the uneasy relationship between HPSR and the mandate of many large donors, partly due to ‘research’ being seen as only indirectly related to interventions (OL2, M1, M3, NG9, B6). Reflecting on issues arising when supporting research by multilateral funders, a senior official with experience across several organizations, explained that “i’’s a little bit pretentious to think that funders that have a very specific mandate and a board that asks them for specific results on their programmatic area can have the freedom to really support whatever is needed in countries for strengthening health systems, policy and strategy in more generic way” (M3). Such issues were even more controversial for donors delivering vertical funding, as HPSR was seen as “a very political way of spending” linked to government priorities which did not always overlap with funder priorities (NG10). In other words, unlike financing for a specific health intervention, research into health policies and health systems was seen by some donors as potentially sensitive for certain governments—an issue we return to in the conclusions.

Of course, possible tensions with the mandate were less of an issue for major research financing organizations per se, who saw it as part of their mission to support HPSR. But even in these cases, supporting HPSR was seen as a side activity, compared to biomedical research, and staff often had to strongly justify their financing in this area. As a senior officer from a major funder explained,

“[HPSR] is hard to pitch to funding committees. This is because the targets are unclear, the methods are so varied, and it’s a different paradigm of research where you’re trying to understand how a system works as opposed to a reductionist view of the world where you have a single intervention or single gene that’s going to do something. The questions seem big and vague and without clear hypotheses. It’s at that blurry line between implementation and what is a government’s responsibility versus what is science.” (NG8)

Overall, these tensions over the mandate of different funders were aptly summarized by an opinion leader with a background in both academia and multilateral donors: “programmatic funders do not appreciate embedding research into programmes, while research funders primarily want innovation [which might not be an aspect of HPSR projects]” (OL2).

One way to overcome such constraints was the identification and operationalization of HPSR activities within organizations’ strategic agendas, taking advantage of their periodic updating. Such statements of organizational strategy could provide cover for project officers to identify potential recipients and design corresponding research programs (M1, NG4, B2). This points to the possible internal organizational struggles to increase prominence for HPSR as a funding priority—this link was most explicit in organizations that saw health systems strengthening as part of their core agenda (NG4).

A related issue that several informants noted was the difficulty in ‘selling’ HPSR projects to the leadership of their organization, domestic stakeholders or other funding partners (NG4, B5). As one informant noted, “we find it quite difficult to explain systems research, and the results are difficult to show. What impact does it really have? How does it change society? We are struggling with these questions and losing extra funding” (B5). These concerns were compounded by the perceived inability to demonstrate concretely value-for-money of HPSR projects (NG9). This scepticism of superiors or collaborators posed some barriers for organizations to engage in HPSR. As an officer in a major non-governmental funder explained,

“there’s perhaps an extra layer of selling or pitching within the organization to stakeholders and leadership levels around the utility and the applicability of research at times. I think there’s quite a bit of scrutiny around health systems and policy research, which is not to say it’s impossible, but it means it takes an extra level of diligence from a program officer point of view” (NG4).

As this quote suggests, mandate constraints appeared to be less an insurmountable barrier, but rather an additional hurdle that project officers had to overcome—a hurdle that took up time and energy to surpass (M3, NG9). For instance, an informant from a multilateral organization providing vertical funding cited struggles within its board and explained:

“I tried to build up support from some board members, especially those who are more systems-oriented. Sometimes this is very difficult to manage because on the other side of the board you may have some strong voices, or even civil society input, who really like to have every single dollar going into the disease-specific work” (M3).

#### Funder capacity and environment

Several informants noted that they had limited capacity to support HPSR projects, whether due to inadequate staff to oversee projects (B5) or the limited amounts of funding available (M1, NG3). A common response was to collaborate with other funders, whether governmental, non-governmental or multilateral, especially where such partners were seen to have the expertise to design and administer effective HPSR projects (B2, B5). Such collaborations could take different forms. For instance, in the UK, public funders (the Medical Research Council and the Economic and Social Research Council), the Department for International Development (now the Foreign, Commonwealth and Development Office) and the Wellcome Trust created the Joint Health Systems Research Initiative to support health systems research in low- and middle-income countries. In other cases, bilateral funders opted to give funds for HPSR purposes to the WHO, who would then administer the related activities (B5, B8).

Relatedly, some informants noted how capacity to support HPSR projects was unevenly distributed within their organizations, partly reflecting a gap between headquarters and field offices. For example, an official in a bilateral funder explained that “a big challenge is making sure that analytical thinking is included in the work of our country offices or even in our broader efforts. So, our collective challenge in some ways is how do we mainstream or ensure that everyone values that this work is part of how we do. Whether we call it HPSR or implementation research or implementation science, it almost doesn’t matter” (B10).

An additional funder-related barrier to supporting HPSR projects—especially affecting bilateral donors—related to how the political environment in which they operate impacted their priorities and the scale and scope of their activities. Governmental or ministerial changes could lead to a reorientation or merger of existing programmes—this in turn affected project officers’ support for certain activities (B2). In other cases, new governments could curtail budgets for foreign or development spending, preventing initiation of new projects (B4). Or some governments might start pursuing policies that limit the scope for conducting research on certain issues: for instance, an official from a bilateral donor recalled that early resistance by some countries to make Covid-19 a priority, so “we just had to adjust our approach based on the politics of the country” (B4).

#### Recipient-related barriers

Turning to barriers to increasing HPSR financing related to recipient institutions, the most common response by informants concerned inadequate research capacities within low- and middle-income countries and the impact of this on the quality of projects. Informants mentioned limited numbers of researchers with the specialized skills necessary to conduct HPSR (M3), the occasional lack of incentives for recipient organizations as they cannot recover high indirect costs (B6), and in some cases inadequate administrative capacity of grantees (NG7, NG8). The latter two issues represent broader constraints on attracting and administering research funding in low-resource settings, and are not specific to HPSR.

However, bilateral and non-governmental donors no longer saw concerns about the capacity of grantees as ‘barriers’ per se (B1, B8, NG1, NG2, NG5), even though they were certainly perceived as such until a few years ago (NG4). Instead, funders increasingly saw capacity strengthening as a key part of their efforts to foster and strengthen research infrastructures within recipient-countries. An informant from a large non-governmental organization recounted a recent case where a prestigious think-tank based in the Global North placed in an excellent bid for a project and was the frontrunner in the selection process, but the grants team decided to instead opt for a consortium of research partners based in the Global South. As the informant explained:

“While some of the work on this project has been very uneven, we are fully committed to this grant because we think we are actually reaching some really strong and dedicated researchers. Part of our agenda then is to say, ‘how can we support the research partners more significantly to help give us the research that we need but also to help strengthening their capabilities and build their capabilities with their students or within their institutions?’” (NG2).

An additional barrier that prevented recipients of HPSR financing to have the desired impact related to their perceived inadequate capacity to translate research findings into actionable items for policymakers (B3, NG4). As one informant opined, “there’s an academic research world and there’s a policy world, and these groups need to communicate with each other and researchers have to make their point why funding for a specific problem is important and policymakers need to understand this” (B1). For example, a senior official at a bilateral funder recalled that their organization tried to connect funded researchers to health policymakers “so that they could share experiences from these two perspectives, and it just didn’t work, because they cannot speak the same language” (B5). In this context, informants noted the importance of developing appropriate fora and formats to communicate research findings and translate them into possible courses of policy action (B9). As one interviewee explained, acting as ‘knowledge brokers’ became part of the job of funders in their supported projects (B9).

However, one opinion leader expressed some scepticism about any simplistic application of the ‘knowledge translation’ agenda:

“Quite a lot of what these initiatives ask for is too simplistic. The answer is, do a three-page memo, improve communication skills, have regular meetings with policymakers and, hey presto, the policymakers are going to take up the research findings. I doubt it. Sometimes if you’re lucky, there is a window of opportunity, a political moment, or trust by a policymaker. But the real world of policymaking is messier. There is need for a more embedded approach: the development of relationships within a context between researchers and not just policymakers, but also managers who make decisions every day and need to use evidence in those decisions. Building those relationships helps to support knowledge translation” (OL1).

The type of engagement proposed by this informant is long term and semi-institutionalized, in ways that commonly exceed the duration of individual projects. Several funders have been considering how to overcome this barrier, and the establishment of and support for networks of researchers, policymakers and advocacy groups—described above—was seen as one intervention to meet the end of improving knowledge translation. In addition, many organizations became actively engaged in helping grantees to overcome such barriers with the provision of practical training and skill development—examples included seminars on how to pitch findings to policymakers, advice on policy writing, or even simple practical measures such as provision of PowerPoint templates to enable streamlined presentations (OL1, B5, NG2, NG4).

#### Barriers inherent to HPSR and its ecosystem

Some informants pointed to barriers to supporting HPSR that are inherent to these research activities. First, several informants noted ‘branding’ issues around HPSR, and some potential confusion over the remits of HPSR and associated terms like ‘implementation science’ or ‘operations research.’ As one opinion leader explained, “there are all of these different terminologies and this is then making things complicated, and not helping the cause” (OL2). Such problems were often encountered in the process of funding research projects, where there were persistent debates around the degree of innovation of HPSR projects, the generalizability of findings, and the diverse research methodologies employed. A senior staffer at a major research funder explained, “We have exactly this problem with implementation science. The response [from funding committees] is always, ‘But that’s implementation and not science.’” (NG8).

In addition, an informant with a background in major multilateral funders expressed the desire for greater WHO involvement and leadership in this area, as WHO engagement would be essential to catalyse cooperation and support from donors. As they opined,

“I don’t think that it should be the Global Fund, or Gavi, or the Gates Foundation to lead the biggest strategic thinking in this area—particularly when zooming out from the purely operational research and towards really addressing the research in terms of policies and strategies. […] When you work in health systems, there is a lot of friction—sometimes coming from vertical funders, sometimes coming from a bilateral perspective. I think that we need to agree on what we mean by health system strengthening and by researching systems and policies. I think the only institution that is very well placed to do this is the WHO. Of course, it should be done in a very technical and global level, but also trying to understand what the specific needs of some of the funders are” (M3).

### Moving forward with HPSR financing

Informants were broadly optimistic about the future of HPSR financing: no respondent anticipated decreases in support, and most expected increases. Nonetheless, we identified several challenges that either held organizations back from more active support for HPSR or entailed higher administrative burdens for project officers involved in supporting such work.

A first key challenge concerned *demonstrating the utility of HPSR and convincing sceptics* who think it is too broad, has excessively long time horizons, or lacks measurable outcomes (B5, NG4). In part, this was due to the complexity of causal pathways: as one informant reflected, “it’s hard to draw a direct line from HPSR to policy change to health outcomes and impact. The logic feels very clear, but the causality is still a bit messy. I think that’s a barrier within our organization that really likes a direct link to outcomes and impact” (NG4). To address such challenges, a major non-governmental donor—in partnership with other non-governmental, bilateral and multilateral funders—is currently working to develop a unified analytical framework to guide their future work on health systems, and this process has embedded in it an HPSR component. Once the framework is developed, one informant explained that “we are going to identify countries and places where we want to work on applying this framework, and then look for a local partner” (NG9). Another interviewee involved in this effort explained that the ongoing effort is to reach a common definition of the problem varies considerably because of how each organization understands their focus on health systems (NG10).

Second, informants noted a scepticism or dissatisfaction vis-à-vis *possible duplication of efforts in HPSR projects*. In this view, HPSR projects illuminated circumstances within a country, but they were commonly accompanied by the background question of how well the research findings travel in other settings. Constituents within the funders or in recipient countries often rejected transfer of findings from one country to another because of cultural, political or other differences. This, in turn, limited the potential for generalizing successful interventions. To overcome this challenge, informants noted that more HPSR work should be done to link cross-country experiences, develop comparative health system analysis, and identify scalable interventions (NG3, NG11, B4). In this context, initiatives like the African Health Observatory were pointed to as possible models for facilitating the cross-border and cross-organization transmission of knowledge and best practices.

A third challenge identified was how to shift away from over-emphasizing problems of inadequate data on health status and health policy in low- and middle-income countries, and—instead—focus on *analysing available data* and developing appropriate policy recommendations. As one senior staffer at a major non-governmental organization explained, “We have pivoted from ‘not enough data is produced in the Global South’ towards ‘not enough data is used in the Global South’. Even what exists and what evidence is out there is not understood, translated, and used” (NG3).

Finally, several informants pointed to the *transformative potential of the Covid-19 pandemic for funder views vis-à-vis HPSR*. As also evident from the funding search presented above, many funders developed HPSR projects that entailed rapid assessments of how countries could respond to the pandemic. Sometimes this was done in the context of ongoing projects, where resources were redirected to support research related to the policy response to Covid (B2). However, as one informant explained,

“the Covid atmosphere is both an accelerator [of HPSR] and a barrier in some ways. There has been intense focus around Covid mitigation and—now—the introduction of the Covid vaccine and therapeutics. So, there’s an attempt to ensure that the introduction of the Covid vaccine has a knock-on effect on strengthening systems, and there’s research on how this can happen. [But] at the same time, there’s an acknowledgment that the introduction of Covid vaccine may need to move faster and won’t be bogged down by the more complex work on systems” (NG4).

Such links between Covid and broader health systems work were expressed by a number of informants—as one explained, “there’s no point focusing on the vaccine if we don’t have the health systems that can deploy the vaccine and support the primary and tertiary care that will be needed to respond to the pandemic,” and—to that end—funding activities had been reoriented towards understanding how to achieve this (NG1). In some cases, Covid-related funding contributed to capacity strengthening too: two interviewees noted support countries with setting up testing capacities and strengthening research institutes (OL2, B1).

Overall, Covid appears to have made the urgency of HPSR clearer and eased some concerns about fit with the mandate within organizations. For example, one informant from a non-governmental funder explained that it used to be case that many grant committees would emphasize the development of vaccines or other innovations but that it was not their mandate to support research “to work out how to actually make this stuff work in the world. But this is slowly changing and developments like Covid are helping that because they illustrate that the fundamental blockers are not the biomedical kit, but the systems in which that kit is put in. It’s now easy to make the argument that we have to research enough to go the final mile that includes health systems and obviously research. It’s much harder as a philosophy, but that argument is easy on a theoretical level” (NG8).

## Discussion

Before discussing the findings of our study, we note two limitations of our analysis. First, although we performed 27 interviews, we are unable to claim representativeness of funding organisations in the global health field. The heterogeneity of organisations with the global health architecture, including the many organisations that play a role in health policy, even if only indirectly or incidentally, makes it very difficult to say where the borders of this community lie. This heterogeneity of focus, but also of framing of global health, is likely to explain some of the differences in how funders approach HPSR issues. Thus, we have previously pointed to at least five ways in which global health may be framed: foreign policy, security, charity, investment, and public health [19]. Health systems and policy research will be seen in differing ways in each of these framings. The same funders may also shift their priorities in response to domestic politics, as shown in research on the effects of change of governments on priorities for development assistance [20]. Beyond that, funders exclusively focused on supporting academic research, like the Wellcome Trust, have different priorities to donors like the Global Fund who may support HPSR in the context of a particular health intervention. Therefore, even though both might allocate funds to HPSR, their motivations and mandates can be very different, which may also shape the nature of the HPSR activities they support. Future work—including using surveys—can elaborate on the different emphases placed by different types of HPSR funders (e.g., research funding bodies like the Wellcome Trust or development funders like the US Agency for International Development), and explore what types of HPSR they are may be more likely to invest in in the future.

Second, our sampling methods led us to funders who are currently involved in some level of HPSR financing, so funders who solely finance biomedical or technological innovations in global health are underrepresented. Even so, to our knowledge, most major funders currently provide some resources to HPSR topics, in line with the emphasis in global policy on health systems strengthening. Future analyses with larger sample sizes would indicate how generalizable our findings were across the wider global-health field.

Our semi-structured interviews reveal a series of critical barriers to expanding HPSR. In some cases, these confirm findings of previous scholarship. However, they also highlight major differences in what researchers and policymakers perceive as key barriers to expanding HPSR. While academic studies tend to highlight recipient-related barriers to increasing HPSR spending [13, 21–24], our informants placed more emphasis on the lack of mandate and capacities of funders themselves. Even so, perhaps the greatest barrier seemed to be the messaging surrounding HPSR: there is no clear narrative that could appeal to funder priorities and the various constituencies in the funder ecosystem. Indeed, informants repeatedly pointed out the difficulties in convincing their organizations’ governing bodies that HPSR leads to lives being saved and concrete improvements in the quality of their interventions.

Overall, our findings point to a growing appreciation of HPSR among funders, even though it is often still seen as an ‘afterthought’ to larger programmatic interventions. A common concern expressed by funders was they did not have a clear mandate or sufficient capacity to pursue it. In this context, the ongoing pandemic presents some opportunities to increase the prominence of HPSR, as it has brought questions of health system capacity and strengthening to the forefront of policy discussions [25]. Relatedly, concerns over the value-for-money of health systems-related interventions remain, and several funders are currently collaborating to develop a framework to ensure uniformity in their approaches and metrics for evaluating performance of their interventions. Yet, such initiatives will depend critically on whether there is sustained support to strengthen capacity in many countries, with realistic timescales which could be a decade or more, accompanied by measures to retain the talent that is being nurtured for the long term, given the global competition for people with the transferable skills that they will acquire.

Another major development is the sea change in how major donors perceive ‘capacity constraints’ for conducting HPSR in low- and middle-income countries. While in the past the lack of such domestic capacities was seen as a reason to not invest in such research or to commission it from institutions in the Global North, current debates on building up domestic capacities has led many donors to reconsider. Echoing calls to ‘decolonize’ development and global health, donors increasingly assume a responsibility to help build up and sustain such skills domestically. This has led to a flurry of funding for cross-country and cross-disciplinary networks that seek to collect cutting-edge evidence to underpin health policies and to strengthen health systems.

But doing so is not without challenges for donors: domestic policymakers can sometimes perceive HPSR as political. Organised health systems are, inherently, redistributional, transferring resources from those who are young, healthy, and rich to those who are old, ill, and poor. This gets to the heart of one of the most important issues differentiating political parties in many countries. In some cases, where minorities, such as indigenous peoples or migrants, are disadvantaged by the existing model of health care, it may also encroach on identity politics, an area of considerable importance in many countries. For example, health outcomes tend to be worse in more fragmented societies, in part because privileged minorities are unwilling to share resources [26]. This can be a very difficult issue to address, especially within a discourse that is increasingly shaped by views about the legacy of colonialism. In a context where many donors want to be seen as neutral vis-à-vis domestic politics, the findings of HPSR may make it harder for them to do so. There may also be more specific factors. Thus, a staffer in a major bilateral global health donor noted recent difficulties in supporting health projects, including HPSR, in Tanzania, given the government’s policy of declaring the country ‘covid-free’ despite evidence to the contrary [27]. The 2005 revision of the International Health Regulations arose, in part, because of concerns about governments in denial about infectious disease in their countries. Of course, such concerns were more prevalent for bilateral and multilateral donors, as they were sensitive to global politics (e.g., missteps by bilateral donors could strain relations between the donor and recipient countries), while non-governmental organizations had fewer such constraints in pursuing their priorities.

## Conclusion

In the aftermath of the emergence of Covid-19, HPSR issues have returned to the foreground of global policy discussions, as countries and donors understand its promise for aiding the development of effective interventions. This does not only relate to pandemic-related priorities, like rolling out Covid-19 vaccines in resource-poor settings, but also to long-standing issues in global health, like actions on the social determinants of health and non-communicable diseases. In short, HPSR serves as a prerequisite for health system strengthening, which can help guide policymakers and donors alike.

But increasing HPSR allocations by donors remains a major challenge. On the one hand, many donors approach their activities in low- and middle-income countries through the lens of their own strategies, priorities and timeframes—supporting and sustaining research on public health, health systems and health policy is a low priority, especially because it does not yield fast results. On the other, despite recent progress, recipient countries often lack infrastructures to conceive, design and implement HPSR, and follow through with the implementation of the relevant findings. Together, these facts contribute to HPSR projects being haphazard and contingent on donor interest.

If HPSR is to build the foundation of effective and efficient health interventions, sustained and sustainable investment in such infrastructures is necessary. Low- and middle-income countries should be in the driver’s seat in this process, as this will ensure that they develop a home-grown culture for HPSR that is supported through domestic resources. External donors certainly have a role to play in this process through funding, advocacy and technical assistance. Yet, these are no substitutes for political commitment to build up HPSR infrastructures and use them to support health policymaking.

## Supplementary Information

**Additional file 1.**

## Data Availability

The interviews were granted under the condition of anonymity. The semi-structured interview questionnaire is presented in Appendix 1.

## Abbreviations

HPSR: Health Policy and Systems Research
WHO: World Health Organization

## References

[1] Kutzin J, Sparkes SP (2016). Health systems strengthening, universal health coverage, health security and resilience. Bull World Health Organ.

[2] Hafner T, Shiffman J (2013). The emergence of global attention to health systems strengthening. Health Policy Plann.

[3] Bcheraoui CE, Weishaar H, Pozo-Martin F (2020). Assessing COVID-19 through the lens of health systems’ preparedness: time for a change. Global Health.

[4] Legido-Quigley H, Asgari N, Teo YY (2020). Are high-performing health systems resilient against the COVID-19 epidemic?. Lancet.

[5] Roder-DeWan S (2020). Health system quality in the time of COVID-19. Lancet Global Health.

[6] Peters DH (2018). Health policy and systems research: the future of the field. Health Res Policy Syst.

[7] Qiu M, Jessani N, Bennett S (2018). Identifying health policy and systems research priorities for the sustainable development goals: social protection for health. Int J Equity Health.

[8] Ghaffar A, Tran N, Røttingen J-A (2014). Health policy and systems research: building momentum and community. Bull World Health Organ.

[9] Kentikelenis A, Ghaffar A, McKee M, Dal Zennaro L, Stuckler D. Global Financing for Health Policy and Systems Research: A Review of Funding Opportunities. Health Policy & Planning. In press. 10.1093/heapol/czac109. 10.1093/heapol/czac109 PMC10019567 36546732

[10] Grépin KA, Pinkstaff CB, Shroff ZC, et al. Donor funding health policy and systems research in low- and middle-income countries: how much, from where and to whom. Health Res Policy Syst 2017;15. 10.1186/s12961-017-0224-6. 10.1186/s12961-017-0224-6 PMC5577666 28854946

[11] Bennett S, Frenk J, Mills A (2018). The evolution of the field of Health Policy and Systems Research and outstanding challenges. Health Res Policy Syst.

[12] Hutchinson E, Balabanova D, McKee M (2019). We need to talk about corruption in Health Systems. Int J Health Policy Manag.

[13] Nabyonga-Orem J, Nanyunja M, Marchal B (2014). The roles and influence of actors in the uptake of evidence: the case of malaria treatment policy change in Uganda. Implement Sci.

[14] Tancred TM, Schleiff M, Peters DH (2016). Health policy and systems research training: global status and recommendations for action. Bull World Health Organ.

[15] Storeng KT (2014). The GAVI Alliance and the ‘Gates approach’ to health system strengthening. Glob Public Health.

[16] WHO Alliance. What is Health Policy and Systems Research and why does it matter ? Alliance Briefing Note. 2007;1.

[17] Ghaffar A, Tran N, Langlois E, et al. Alliance for Health Policy and Systems Research: aims, achievements and ambitions. Public Health Research Pr 2017;27. 10.17061/phrp2711703. 10.17061/phrp2711703 28243669

[18] Kalibala S, Woelk GB, Gloyd S (2016). Experiences in implementation and publication of operations research interventions: gaps and a way forward. J Int AIDS Soc.

[19] Stuckler D, McKee M (2008). Five metaphors about global-health policy. The Lancet.

[20] Greene ZD, Licht AA (2018). Domestic politics and changes in Foreign Aid Allocation: the role of Party Preferences. Polit Res Q.

[21] Khan MS, Meghani A, Liverani M (2018). How do external donors influence national health policy processes? Experiences of domestic policy actors in Cambodia and Pakistan. Health Policy Plan.

[22] Mutero CM, Kramer RA, Paul C (2014). Factors influencing malaria control policy-making in Kenya, Uganda and Tanzania. Malar J.

[23] Ezenwaka U, Mbachu C, Etiaba E (2020). Integrating evidence from research into decision-making for controlling endemic tropical diseases in South East Nigeria: perceptions of producers and users of evidence on barriers and solutions. Health Res Policy Syst.

[24] Hennink M, Stephenson R (2005). Using research to inform health policy: barriers and strategies in developing countries. J Health Commun.

[25] WHO. Drawing light from the pandemic: A new strategy for health and sustainable development. World Health Organization. 2021.https://www.euro.who.int/en/health-topics/health-policy/european-programme-of-work/pan-european-commission-on-health-and-sustainable-development/publications/drawing-light-from-the-pandemic-a-new-strategy-for-health-and-sustainable-development-2021 (Accessed 10 Sept 2021).

[26] Powell-Jackson T, Basu S, Balabanova D (2011). Democracy and growth in divided societies: a health-inequality trap?. Soc Sci Med.

[27] Buguzi S (2021). Covid-19: counting the cost of denial in Tanzania. BMJ.

