# Supplementary material for: Donor support for Health Policy and Systems Research: barriers to financing and opportunities for overcoming them
Source: Global Health. 2022 Dec 23;18:106. doi: 10.1186/s12992-022-00896-4 (PMC9782264; doi:10.1186/s12992-022-00896-4)
Supplement: Supplementary file 1 — Additional file 1. [file 12992_2022_896_MOESM1_ESM.docx]

# Web Appendix 1. Questionnaire for semi-structured interviews

## PART 1: Introduction

### Opening

- Brief comment on the remit of the project and intended outcomes
- Emphasize anonymity in any published outputs, and citations will be attributed to generic positions (e.g., “senior grants officer in a major multilateral organization”)
- Clarification about terminology: We use HPSR, although there are some overlaps with “implementation research” or “operation research”
- But HPSR is broader in that it aims to generate new and reliable knowledge on health policy and systems, and promotes its application in health interventions — not just tied to research that is of sole use to a specific intervention
- Opening question: Motivations for financing HPSR activities
- Does [your organization] support research into health systems and policy, and how?

## PART 2a: For organizations that consistently support HPSR

### Deciding on HPSR financing

- Are stand-alone grants provided for HPSR, or is HPSR embedded in bigger projects?
- How do you select recipients for research projects on health systems and policy?
- Are HPSR allocations a considered favourably within [your organization]?

### Priorities in HPSR financing

- Are there specific areas that are targeted by [your organization’s] HPSR funding?
  - New data collection? If so, how do you use the data?
  - Local research capacity strengthening projects?
  - Knowledge translation projects for local policymakers and advocacy groups?

### Possible barriers for increasing HPSR financing

- Have you encountered any major barriers in developing or administering HPSR projects?
- Are any of the following issues keeping you back from increasing financing?
  - Lack of capacity of local or international partners to conduct HPSR
  - Lack of trust by policymakers in HPSR findings
  - Lack of interest by policymakers in HPSR findings
  - Insufficient quality of HPSR projects

### Examples

- Have any HPSR projects generated concrete advantages for [your organization]?
- Can you recall some examples of HPSR projects that went exceptionally well/badly? Why do you think that was the case?
- Have you encountered cases where HPSR would have been helpful to meet the objectives of a project, but was not conducted as it was outside of its scope or beyond available financing?
- Have lessons from these projects been built into overall organizational practices or funding approaches?

## PART 2b: For organizations that inconsistently support HPSR or do not support it at all

### Deciding on HPSR financing

- What has kept [your organization] from investing in HPSR projects?
- Are HPSR allocations a considered favourably within [your organization]? Are there constituencies advocating for such projects, or resisting them?

### Possible barriers for increasing HPSR financing

- Have you encountered any major challenges in HPSR projects? Can you provide examples?
- Are any of the following issues keeping you back from increasing financing?
  - Lack of capacity of local or international partners to conduct HSPR
  - Lack of trust by policymakers in HPSR findings
  - Lack of interest by policymakers in HPSR findings
  - Insufficient quality of HPSR projects

### Examples

- Can you recall some examples of HPSR projects that went exceptionally well/badly? Why do you think that was the case?
- Have you encountered cases where HPSR would have been helpful to meet the objectives of a project, but was not conducted as it was outside of its scope or beyond available financing?
- Have lessons from these projects been built into overall organizational practices or funding approaches?

## PART 3: Closing

### Future

- Does [your organization] have any plans to increase or cut HPSR allocations? Why?

### Closing off

- Could you recommend other experts or policymakers we should interview on HPSR financing issues?
